# Supplementary material for: Evolution Analysis of Free Fatty Acids and Aroma-Active Compounds during Tallow Oxidation
Source: Molecules. 2022 Jan 6;27(2):352. doi: 10.3390/molecules27020352 (PMC8779770; doi:10.3390/molecules27020352)
Supplement: Supplementary file 1 [file molecules-27-00352-s001.zip › molecules-1530105-supplementary.pdf]

Table S1. Standard curves of aroma−active compounds.

Compound	Standard Curve	R^2	LOD (mg/kg)	LOQ (mg/kg)
Hexanal	y = 0.69487x − 0.07866	0.99964	1.19	3.97
1-Penten-3-ol	y = 0.09988x + 0.00404	0.99621	0.58	1.94
Heptanal	y = 0.14389x − 0.00251	0.99865	0.50	1.65
D-Limonene	y = 0.56831x − 0.01224	0.99869	0.49	1.63
(*E*)-2-Hexenal	y = 0.15672x + 0.00033	0.99796	0.21	0.69
2-pentylfuran	y = 0.58169x − 0.01897	0.99894	0.08	0.25
1-Pentanol	y = 0.15509x − 0.00226	0.99851	0.40	1.34
2-Octanone	y = 0.28257x − 0.00668	0.99875	0.13	0.43
Octanal	y = 0.21745x − 0.01109	0.9982	0.91	3.03
(*E*)-2-Heptenal	y = 0.23307x − 0.00256	0.99962	0.32	1.06
2-Nonanone	y = 0.52404x − 0.04584	0.99407	0.53	1.77
Nonanal	y = 0.843x − 0.09298	0.99326	0.39	1.30
(*E*)-2-Octenal	y = 0.38068x − 0.03393	0.99383	0.11	0.38
1-Octen-3-ol	y = 0.31183x − 0.00656	0.9994	0.55	1.83
1-Heptanol	y = 0.32085x − 0.0129	0.99848	0.10	0.35
(*E,E*)-2,4-Heptadienal	y = 0.28538x + 0.11322	0.99483	0.17	0.57
Decanal	y = 0.29125x − 0.02488	0.99718	0.55	1.83
Benzaldehyde	y = 0.6488x − 0.01042	0.99935	1.16	3.88
(*E*)-2-Nonenal	y = 0.37671x − 0.01913	0.99796	0.68	2.25
Propanoic acid	y = 0.13163x + 0.02013	0.99187	0.24	0.81
1-Octanol	y = 0.33019x − 0.01765	0.99833	0.69	2.31
Gamma-Valerolactone	y = 0.17123x − 0.00477	0.99842	0.32	1.08
Butanoic acid	y = 0.32623x − 0.02181	0.99786	0.33	1.10
Gamma-Hexalactone	y = 0.31295x + 0.00503	0.99687	0.10	0.33
Pentanoic acid	y = 0.14903x − 0.00384	0.99976	0.68	2.28
Gamma-Heptalactone	y = 0.48038x − 0.02853	0.99685	0.16	0.52
(*E,E*)-2,4-Decadienal	y = 0.31049x − 0.0006875	0.99799	0.08	0.27
Hexanoic acid	y = 0.28146x − 0.00875	0.99961	0.15	0.51
Gamma-Octalactone	y = 0.66578x + 0.03967	0.99762	0.25	0.84
Heptanoic acid	y = 1.03952x − 0.19492	0.9739	0.56	1.87
Octanoic acid	y = 0.37274x − 0.05133	0.99974	1.72	5.75
Nonanoic acid	y = 0.38859x − 0.05638	0.99984	0.23	0.76
n-Decanoic acid	y = 0.70366x − 0.13311	0.99938	0.70	2.35
Dodecanoic acid	y = 0.83913x − 0.21726	0.99936	1.04	3.47
Tetradecanoic acid	y = 0.62483x + 0.1651	0.99936	2.63	8.77

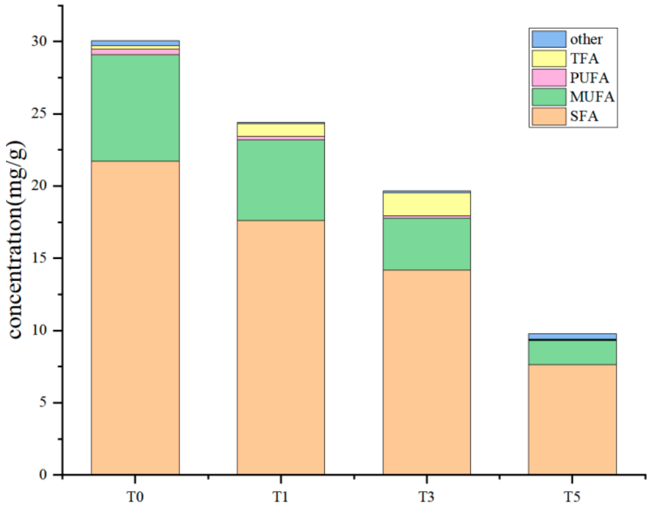

Figure S1. FFAs concentration histogram (SFA: saturated fatty acid, MUFA: monounsaturated fatty acid, PUFA: polyunsaturated fatty acid, TFA: trans fatty acid).
